# Influence of Stabilization Additive on Rheological, Thermal and Mechanical Properties of Recycled Polypropylene

**DOI:** 10.3390/polym14245438

**Published:** 2022-12-12

**Authors:** Mohor Mihelčič, Alen Oseli, Miroslav Huskić, Lidija Slemenik Perše

**Affiliations:** 1 Faculty of Mechanical Engineering, University of Ljubljana, Aškerčeva Ulica 6, 1000 Ljubljana, Slovenia; 2 Faculty of Polymer Technology, Ozare 19, 2380 Slovenj Gradec, Slovenia

**Keywords:** recycling, polypropylene, stabilization additive, rheological properties, thermal properties, mechanical properties

## Abstract

To decrease the amount of plastic waste, the use of recycling techniques become a necessity. However, numerous recycling cycles result in the mechanical, thermal, and chemical degradation of the polymer, which leads to an inefficient use of recycled polymers for the production of plastic products. In this study, the effects of recycling and the improvement of polymer performance with the incorporation of an additive into recycled polypropylene was studied by spectroscopic, rheological, optical, and mechanical characterization techniques. The results showed that after 20 recycling steps of mechanical processing of polypropylene, the main degradation processes of polypropylene are chain scission of polymer chains and oxidation, which can be improved by the addition of a stabilizing additive. It was shown that a small amount of an additive significantly improves the properties of the recycled polypropylene up to the 20th reprocessing cycle. The use of an additive improves the rheological properties of the recycled melt, surface properties, and time-dependent mechanical properties of solid polypropylene since it was shown that the additive acts as a hardener and additionally crosslinks the recycled polymer chains.

## 1. Introduction

In today’s world, with the growing global prosperity, plastics have become a major part of our everyday life. The raw materials that are necessary for the production of such large quantities of plastic are, however, limited. Moreover, an annual increase in plastic waste made from nonrenewable sources has a negative impact on the environment [1]. The easiest way to decrease the environmental impact is mechanical recycling, which enables the transformation of plastic waste back to useful products. However, studies show that less than 15% of plastics in the world have been recycled. One of the polymers, which is easy to recycle, is polypropylene. It is the world’s second-most produced thermoplastic polymer. Due to its good mechanical properties, it is widely used in the automotive, aerospace, and packaging industries; furniture; textiles, etc. [2,3,4,5] Moreover, it is highly adaptive to a wide range of different applications and types of production; therefore, PP has maintained a significant market share compared to other polymers. The problem with PP and most of the thermoplastic polymers is a deterioration of their mechanical properties caused by polymer chain degradation induced by mechanical reprocessing.

The degradation of polyolefins could be, in general, divided into three types: mechanical degradation, thermal degradation, and thermal oxidative degradation [6,7]. The changes due to the recycling process are visible through continued deterioration of physical properties, such as molecular structure, viscosity, degree of crystallinity, or Young’s modulus [8,9,10,11]. It is known that thermo-mechanical reprocessing causes chain scission of the PP, leading to a decrease in thermal stability, melting temperature, viscosity, and viscoelastic properties with increasing reprocessing cycles [12,13,14]. One of the solutions for maintaining or improving the mechanical properties during recycling is the addition of various additives to recycled plastics. Similarly, the additives that can improve several properties could be included already to the virgin polymers.

The research on the improvement of polypropylene properties is vast; however, many studies have been performed on plastic additives focusing mostly on mineral-based (reinforcing fillers), i.e., CaCO_3_, talc, kaolin, mica [15] [16,17,18], as well as antioxidant stabilizers [19,20], and others. One of the useful additives is also CaO, which is a water-absorbing additive and neutralizes acidity [21]. Among various polymers, the polypropylene is slightly more susceptible to attack by strong oxidizing agents. It is known that the addition of antioxidants protects polymers at their first processing cycle, while further research of antioxidant stability during further repetitive extrusion cycles has rarely been studied. It has been reported that some hindered amines (HALS), and phenolic and phosphorous compounds, such as Tinuvin, Irganox, or Irgafos, could be used to improve the antioxidant properties of PP stability [19]. Among many characterization methods, ATR-IR spectrometry has already become a routine analysis for the quantitative characterization of antioxidative PP stability [22]. The studies reported in the literature are mainly focused on the rheological and mechanical properties of neat or recycled polymeric materials; however, according to our knowledge, there is a lack of investigations on the degradative effects in stabilized recycled polypropylene.

For this reason, our study focuses on the effect of recycling on rheological properties (indicating structural changes in polymer melt), thermal properties (related to phase transitions and crystallinity), and mechanical properties (associated with the practical use of solid polymer products). Moreover, the improvement of polymer performance with the incorporation of an additive into recycled polypropylene was studied with polypropylene, which was recycled by extensive mechanical processing.

## 2. Materials and Methods

### 2.1. Materials and Sample Preparation

In this study, the polypropylene (PP) homopolymer, Buplene 6331, purchased by Lukoil (LUKOIL Bulgaria Ltd., Sofia, Bulgaria; MFI of 8–16 g/10 min at 230 °C and 2.16 kg) was used. Commercially available Recyclobyk 4371 additive (provided by BYK-Chemie GmbH, Wesel, Germany) was used for the stabilization of recycled PP. The additive is usually used for the stabilization of polyolefin blends, i.e., recyclates from the automotive industry and the packaging industry; fiber-reinforced plastics with recyclates; and for the reduction of VOC and odors. The additive was purchased in a pellet form as a mixture of antioxidants and co-stabilizers. According to the producer, the additive helps to improve the mechanical properties of recycled polymer and helps to neutralize acids that may be present in the polymer from previous processing and use.

Mechanical recycling of PP samples was performed by using a co-rotating twin-screw extruder, Polylab PTW 16/40 OS (Thermo Scientific, Karlsruhe, Germany), with the screw speed set at 90 rpm. The temperatures on the ten heaters of the extruder were set ascending with the temperature of the first heater at 150 °C, while the last heater was set to a process temperature (205 °C).

After drying at 90 °C for 4 h, the virgin (as received) PP was reprocessed 10 times into 2-mm-long granules. After the 10th cycle, the granulated rPP batch was halved into two batches. One-half of the batch was characterized as the batch without additive, while 0.75% wt. of Recyclobyk 4371 additive was added to the second half in order to improve the mechanical properties of the recycled material. The recycled batches without (denoted as rPP) and with additive (denoted as rPP_a) were extruded and regranulated for ten more cycles (Table 1). The additive was introduced to PP, which was recycled for 10 times since our intention was to test if a degraded PP could be re-stabilized.

Test bars (L = 60, w = 10, h = 1 mm) for the dynamic mechanical analysis (DMA) were injection-molded on a Haake MiniJet II (Thermo Scientific, Karlsruhe, Germany) injection-molding machine. Samples were prepared by using the processing parameters presented in Table 2.

DMA test bars were used for all measurements, except for rheological characterization, where virgin or extruded granules were directly placed in the sensor system. Prior to creep and DMA testing, the samples were thermally annealed in order to erase the residual stresses. The annealing was performed at 110 °C for 2 h followed by cooling to room temperature at 0.1 °C/min.

### 2.2. Methods

#### 2.2.1. ATR-IR Characterization

ATR-IR measurements were performed on a Perkin Elmer, Spectrum 65 (Waltham, MA, USA) FTIR spectrometer, equipped with a single reflection diamond crystal at room temperature. ATR-IR spectra were recorded in a range from 4000 to 600 cm^−1^ at 64 scans per spectrum with a resolution of 4 cm^−1^.

#### 2.2.2. Polarized Optical Microscopy

The phase transformation of PP was investigated using a Carl Zeiss AxioScope polarizing optical microscope (Carl Zeiss, Braunschweig, Germany) equipped with a 200× objective and a Linkam TS600 Heating Stage. For optical characterization, the samples were prepared as 10-μm-thick films using a microtome. The films were heated between a glass slide and coverslip to 200 °C and kept at constant temperature for 5 min. Subsequently, the samples were rapidly cooled to 130 °C for isothermal crystallization.

#### 2.2.3. Thermal Properties

Differential Scanning Calorimetry (DSC) measurements of the samples were carried out on TA Instruments DSC Q2500 (TA Instruments, New Castle, DE, USA). Samples weighing 6 ± 1 mg were cut out from injection-molded DMA bars and placed into aluminium cups. Measurements were performed from −50 °C to 250 °C at a rate of 10 K min^−1^ followed by a 5 min range of constant temperature at 250 °C and cooling to −50 °C at a rate of 10 K min^−1^. At the lowest temperature, the samples were exposed to a constant temperature again for 5 min; in this case, at −50 °C. Second heating, which was also used for the analysis, was conducted to obtain results independent from the thermal and mechanical history of the samples. The inert atmosphere with a nitrogen gas flow of 50 mL·min^−1^ was provided during the measurements. All measurements were repeated 3 times and the average values were used for the analyses. The results of the DSC tests were used to determine the transition temperatures and the degree of crystallinity (***X_C_***) as:

(1) $$X_{C}=\frac{\Delta H_{m}}{\Delta H_{m}^{0}},$$

where $\Delta H_{m}$ is the experimentally-determined enthalpy of melting and $\Delta H_{m}^{0}$ is the theoretical value of the melting enthalpy of 100% crystalline PP (207 J/g) [23].

#### 2.2.4. Rheological Properties

The rheological characterization of PP and rPP polymer melts were conducted at a constant temperature of 190 °C using the Anton Paar MCR 302 rotational rheometer (Anton Paar, Graz, Austria) equipped with parallel-plate geometry (plate diameter of 25 mm with a 1 mm gap) in inert nitrogen atmosphere. For the analysis, the samples in granulated form were directly placed on the lower plate of the sensor system. The viscoelastic properties (storage modulus—G’ and loss—G’’) of the studied polymer melts were determined using amplitude and frequency oscillatory tests. The frequency sweep experiments were carried out from 100 to 0.01 Hz using a constant strain of 5%, which was within the linear viscoelastic region (LVR) previously determined from amplitude tests. The average molecular weight (***M_w_***) and polydispersity index (PDI = ***M_w_***/***M_n_***) were determined from rheological frequency tests using the ***Rheocompass*** software (v.1.30).

#### 2.2.5. Mechanical Characterization

##### Nanoindentation

The hardness and elastic modulus of the surface of injection-molded samples were determined using a Nanoindenter G200 XP instrument manufactured by Agilent (Santa Clara, CA, USA). For the measurements, a standard three-sided pyramidal Berkovich probe was used. The mechanical properties were determined by using the CSM (Continuous Stiffness Measurement) [24] method with a tip oscillation frequency of 45 Hz and a 2 nm harmonic amplitude. All measurements were conducted at room temperature. For virgin and recycled PP material, 36 indentations on each sample were performed with a 100 µm distance between the indentations, which enabled the exclusion of interaction effects. The measuring depth was 2000 nm and the average values between 800 and 1800 nm in depth were used in the presented results.

##### Time-Dependent Measurements

Creep tests were conducted on the Mars Haake II Rheometer (Thermo Scientific, Karlsruhe, Germany) equipped with a controlled-temperature test chamber and solids-clamping tool. Shear creep compliance ***J(t)*** tests were carried out in a linear viscoelastic region at a constant shear stress, τ, of 0.01 MPa. Measurements were performed in the segmental form at nine different temperatures between 20 and 100 °C with a step of 10 °C. A time-temperature superposition (tTS) principle was used for the characterization of the time-dependent behavior of rPP, with and without additive, for the improvement of mechanical properties (for a detailed analysis protocol, see Ref. [25]).

##### Dynamic Mechanical Thermal Analysis (DMTA)

For all the mechanical characterizations, at least three samples (L = 50, w = 10, h = 1 mm) for each recycling set were tested in the temperature range from −18 °C to 110 °C, with a heating rate of 3 °C/min and an oscillation frequency of 1 Hz. The shear strain during the measurements was logarithmically increased from 0.02 to 0.1%. DMTA tests were performed using the Anton Paar MCR 702 (Anton Paar, Graz, Austria). The results enabled the determination of the glass transition temperature (*T_g_*), which was determined at the temperature where tan, δ, exhibited a maximum value.

## 3. Results and Discussion

### 3.1. ATR-IR Characterization

**Figure 1:**
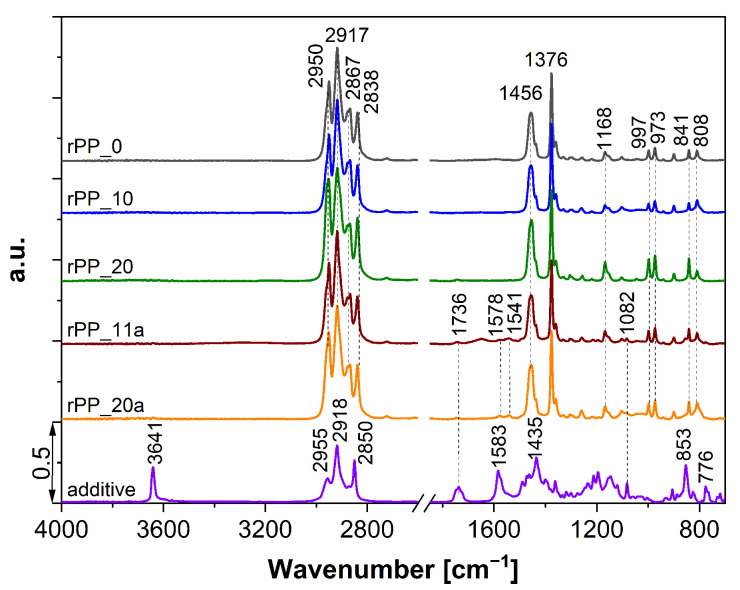
ATR-IR spectra of virgin PP, recycled with (rPP_a) and without (rPP) additive.

IR spectroscopy is one of the most common methods for studying the degradation and stabilization of PP under various conditions [26,27]. The degradation behavior of recycled PP with and without additive was studied using ATR-IR spectroscopy, and the results are shown in Figure 1. The virgin PP (rPP_0) shows characteristic groups in the polymer chain with CH_3_ stretching bands at 2950 and 2867 cm^−1^, and CH_2_ stretching bands at 2917 and 2838 cm^−1^ [28]. The CH_3_ banding vibration peak can be assigned to asymmetric δ_as_(C-H) at 1456 cm^−1^ and the CH_2_ banding vibration to symmetric deformation δ_s_(C-H) at 1376 cm^−1^ (Figure 1). The bands at 841, 997, and 1168 cm^−1^ are ascribed to vibrations characteristic of isotactic polypropylene [29]. Moreover, the shoulder peak at 2960 cm^−1^ (ν_as_(methyl group -CH_3_) increases with the increasing number of recycles, and is much more pronounced at rPP_20 (without additive). The findings suggest that the bands, 2960 cm^−1^ and 2837 cm^−1^, are the recycling-sensitive bands, which were already confirmed by other authors [30]. This could be an identification of the chains scission of PP during multiple processing cycles.

The ATR-IR spectra of the additive (Recyclobyk 4731), which is a mixture of antioxidants and co-stabilizers, showed a peak at 3641 cm^−1^, which can be assigned to isolated OH groups present at the surface of the CaO solid and peak at 776 cm^−1^, possibly due to the Ca–O bonds. The band at 1583 cm^−1^ corresponds to the carboxylate bond (–COO^−^) and it disappeared after the first recycling using an additive (rPP_11a). New double bands at 1578 and 1541 cm^−1^ were observed for rPP_11a and rPP_20a, indicating the formation of carboxylate groups due to the addition of the additive.

### 3.2. Polarized Optical Microscopy

**Figure 2:**
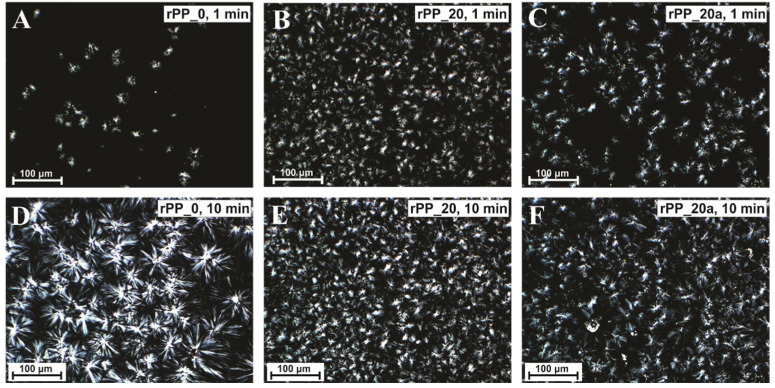
Polarizing optical microscopy of the nucleation and growth processes of virgin PP—rPP_0 (**A**,**D**), rPP_20 (**B**,**E**), and rPP_20a (**C**,**F**) after isothermal crystallization at 130 °C for 1 min (**A**–**C**) and 10 min (**D**–**F**).

The polarized optical microscopy (POM) analysis is a well-established method to determine crystallization behavior. The effects of recycling PP with and without additive were studied isothermally at 130 °C. The results (Figure 2) show that the formation of crystals was the slowest for the virgin PP (rPP_0). The first individual spherulites appeared after 1 min (Figure 2A) while (Figure 2D) the crystallization process was finished after 10 min, resulting in a small amount of crystals of relatively large size. On the other hand, the crystallization rate with an increasing number of recycling cycles increases (Figure 2B) while the crystal size decreases. This is a consequence of the shorter polymeric chains resulting from the degradation during the recycling process [11]. A high number of recycles leads to densely-packed crystallites, with the boundaries hard to distinguish. High nuclei density hinders crystals to grow freely; moreover, the crystals are much smaller than those observed for the virgin polypropylene. The addition of an additive beneficially influences the growth of crystallites, also at higher processing cycles, as larger crystallites formed in rPP_20a compared to rPP_20 without additive (Figure 2C). During the observation of the crystallization process with optical microscopy, it was observed that the formation of crystals at rPP with additive occurs sooner compared to rPP without additive. In order to further make more detailed investigations, DSC measurements were performed.

### 3.3. Thermal Properties

**Figure 3:**
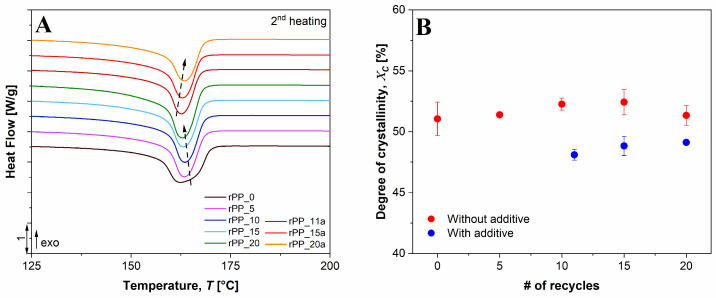
(**A**) DSC thermograms of melting peaks from the 2nd heating cycle of virgin PP, recycled PP without and with additive, and (**B**) the degree of crystallinity, determined by 2nd heating.

The DSC is an important characterization method to determine the effects of recycling on the thermal behavior of semi-crystalline virgin and recycled PP. Additionally, the results enable the determination of the changes arising from the addition of the additive to recycled PP. The first heating run gives information on the material’s thermal properties after the melt processing, including the mechanical and thermal history (influence of processing, crystallinity, ageing, heat treatment, etc.) For the determination of the crystallization temperature (***T_c_***), the studied samples were investigated under controlled cooling. With second heating (third run), the obtained data provide information on the material’s glass transition (***T_g_***) and its melting temperature (***T_m_***), independent of the history. The DSC analysis of virgin PP, recycled with additive and recycled without additive, is presented in Figure 3 and summarized in Table 3. The results of the 2nd DSC heating (Figure 3A) show that the melting temperature (***T_m_***) of virgin PP (rPP_0) was 162.0 °C. As the number of recycles increased, the ***T_m_*** increased up to the 10th cycle, while further increasing of the recycling process decreased the ***T_m_***. The addition of an additive to rPP_10 slightly decreased the ***T_m_*** for rPP_11a, while further increasing of the recycles led to an increase of the ***T_m_***. However, the measured values of ***T_c_*** and ***T_m_*** were within the experimental error, calculated from three repeated measurements.

For the enthalpy of PP melting, the results (Table 3) show a constant upward trend with an increased number of recycles, while the addition of the additive to rPP_10 decreased the enthalpy sharply (from 108.2 J/g for rPP_10 to 99.6 J/g for rPP_11a). A similar result was observed for the determination of the degree of crystallinity (Figure 3B). With an increasing number of recycles, the crystallinity of additive-free samples increased from 51.1% to 51.3%. However, after the additive was added to rPP_10, the ***X_C_*** first decreased by ~8%, while with a further nine recycles, the ***X_C_*** increased only by ~0.2%. The increased crystallinity of the recycled PP could be attributed to the molecular weight reduction and smaller entanglements of chains due to several thermal-reprocessing cycles [31]. This can also be observed from the DSC measurements where the peaks with the same shape and size are only shifted, meaning that the changes are only due to the chain scission and not to changes in the molecular size of the formed structure. The lower molecular weight of PP contributes to a higher mobility of the chains, resulting in higher crystallinity; however, more imperfections in the resulting crystals could be expected. As mentioned before, such imperfections are mainly due to the formation of free radicals during the rupture of the molecular chains. These results are in agreement with the POM analysis, where a different kinetic rate between the virgin and recycled PP samples was observed.

The increase in the degree of crystallinity can also be explained by the fact that macromolecules with lower molecular weight act as nucleating agents, which enhance the crystallization of semicrystalline polymers by enabling the folding of the chains and building bigger crystal structures. Correspondingly, as a result of the decreased molecular weight, the crystallization temperature increases with the reprocessing cycles.

**Figure 4:**
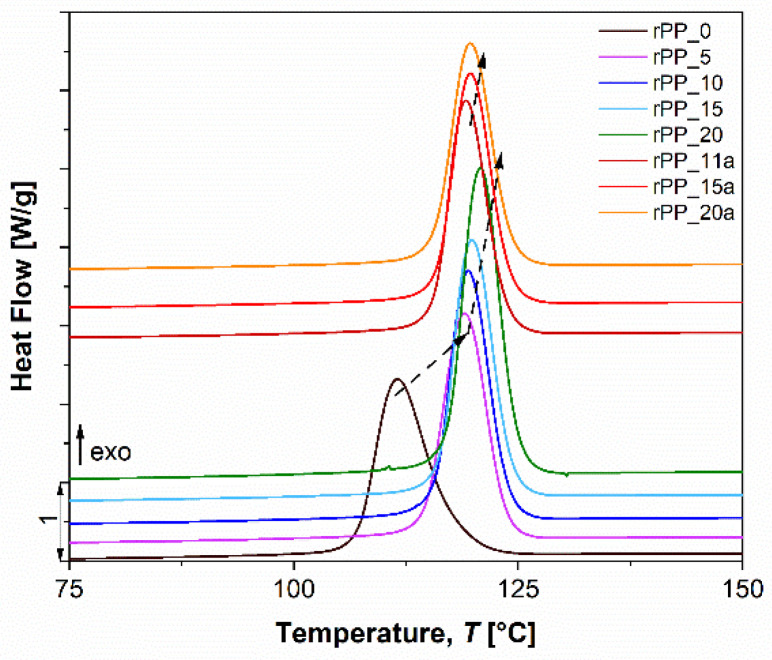
DSC thermograms of crystallization peaks of virgin PP, and recycled PP without and with additive.

Figure 4 shows a main exothermic peak at 112.9 °C, corresponding to the crystallization of virgin PP. After recycling virgin PP 20 times (rPP_20), an increase in crystallization temperature (***T_C_***) of 9.1 °C was observed for the sample without additive, while the increase for rPP with additive (rPP_20a) was 8 °C. These results show that thermal recycling promotes the formation of crystalline phases in the rPP samples.

### 3.4. Rheological Properties

**Figure 5:**
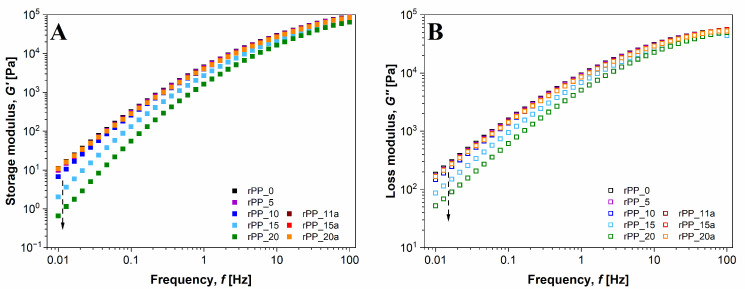
Frequency dependency of (**A**) storage modulus ***G’*** and (**B**) loss modulus ***G’’*** of virgin PP, recycled PP without and with additive depending on the processing cycle (at 190 °C).

The viscoelastic measurements are well-established and practical experimental methods for melt characterization. The rheological behavior, presented by storage modulus (***G’***), loss modulus (***G”***), and complex viscosity (η*) of virgin and recycled PP with and without additive have been studied. The results of viscoelastic (***G’*** and ***G’’***) properties in the linear viscoelastic region (LVR) at different frequencies (Figure 5) show that the values of both moduli (***G’*** and ***G’***) increased with an increasing frequency of oscillation with ***G’’*** prevailing in the range of low frequencies. Moreover, the moduli of the recycled PP without additive decreased with increasing reprocessing cycles, indicating a degradation of the structure. After a higher number of recycling steps, PP exhibited a more liquid-like character due to chain-breaking and a reduction of molecular weight.

On the other hand, a major improvement in viscoelastic properties occurred when the additive was added to PP, which had already been recycled 10 times. Compared to virgin PP, the ***G’*** and ***G’’*** of the 10-times-recycled PP with the addition of additive increased in the whole frequency range examined. This indicates that the additive propagated crosslinking between polymer chains with chain scissions, which improved viscoelastic behavior. The results suggest that the additive limits the mobility of the polymer chain and thus, reinforces the internal network structure of rPP.

**Figure 6:**
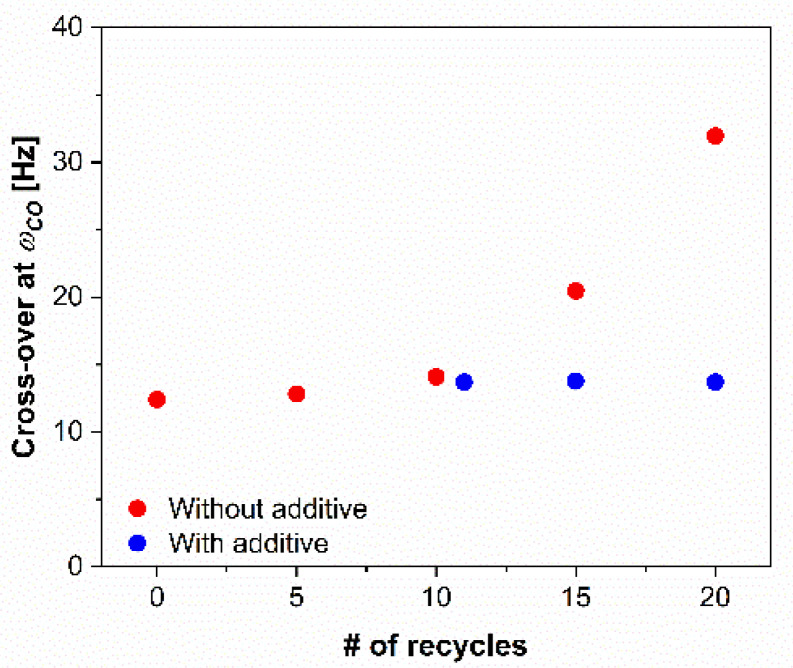
Crossover frequency, *ω*_*co*_, of virgin PP, and recycled PP without and with additive, depending on the processing cycle.

If we compare the frequency dependency of ***G’*** and ***G’’***, respectively, we can investigate the point where the moduli have the same value. At this point, the behavior of the material changes; in our case, from liquid-like in the low frequency range to solid-like at higher frequencies. Figure 6 shows the intersection points between ***G’*** and ***G’’*** with respect to the frequency. This point is called the crossover modulus point (***G*** = ***G’*** = ***G’’***) and the frequency at which the moduli are the same is called crossover frequency (*ω*_*co*_). If all samples are compared, it becomes clear that for rPP samples without an additive, the crossover point of ***G’*** and ***G’’*** shift towards higher frequencies where the relaxation times of the molecules are lower. This is characteristic of shorter molecules, and it reflects the chain scission during the extensive recycling process. On the other hand, when the additive was added to the sample, recycled for 10 times, the crossover frequency remained the same regardless of the number of further processing cycles.

**Figure 7:**
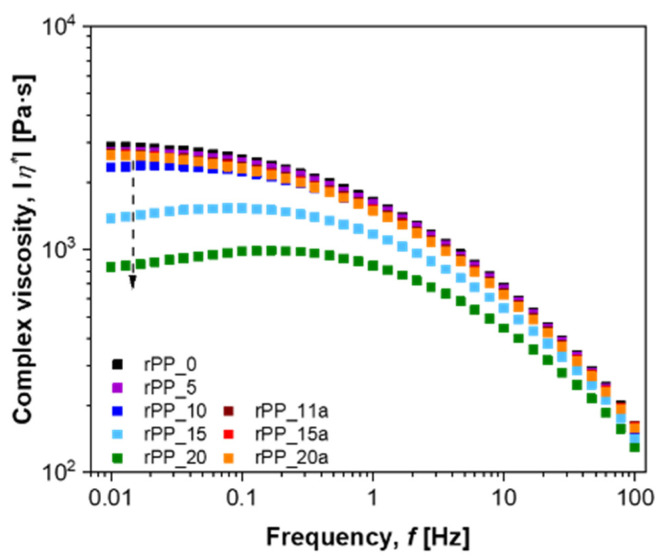
Dynamic rheological behavior of virgin PP, recycled PP without and with additive, complex viscosity, measured at 190 °C.

In addition to the storage modulus (***G***′) and the loss modulus (***G***″), the complex viscosity is also an important parameter that provides information about the viscous or elastic effects. As presented in Figure 7, the complex viscosity for all rPP samples decreased with increasing frequency, showing a typical shear-thinning behavior [10]. The zero-shear viscosity (*η*_0_), i.e., the constant melt viscosity at low frequencies, decreased with the number of recycling cycles of rPP without additive. This can be correlated to the decreased molecular weight and polymer chain length where fewer entanglements between chains and intermolecular interactions occurred [13,32,33]. The shear-thinning behavior of rPP recycled for 20 times without additive was the least pronounced with the lowest zero-shear viscosity of 845 Pa·s, which is almost four times lower than the *η*_0_ of virgin PP. The transition between the Newtonian and the shear-thinning region of the viscosity curve was smoother and occurred at lower frequencies. Furthermore, the addition of an additive increases the entanglements of the molecules due to the promotion of crosslinking of broken polymer chains, which were reflected in a higher *η*_0_ for recycled samples compared to the virgin PP. Further recycling (up to 10 more times) did not affect the *η*_0_.

**Figure 8:**
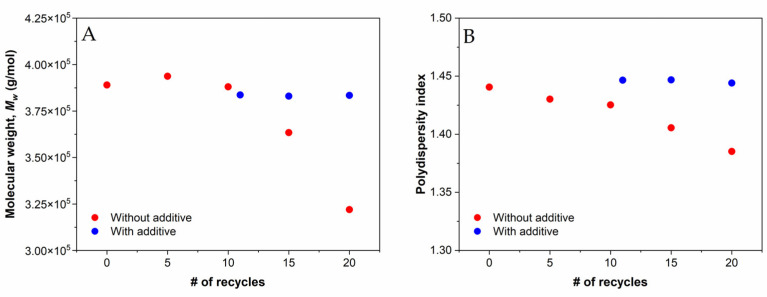
(**A**) Molecular weight distribution and (**B**) polydispersity of virgin PP, recycled PP without and with additive, respectively.

The changes in the average molecular weight (***M_w_***) and polydispersity index (PDI) with an increasing number of recycling steps for PP without and with additive (Figure 8AB) were obtained from the rheological measurements of frequency sweep analysis [34], which has been reported in the literature to be in good agreement with the results of gel permeation chromatography (GPC), the most commonly used technique to determine ***M_w_*** [35]. It has been observed that the peak of ***M_w_*** shifts to lower values with increasing recycling steps, which can be attributed to degradation and chain scission [36]. A significant decrease in ***M_w_*** was observed after the 10th recycling step without the addition of the additive. Furthermore, our results show that the ***M_w_*** did not decrease further after the addition of the additive. The results are in very good agreement with the rheological characterization data, which also show a significant decrease in complex viscosity after the 10th recycling step for rPP without additive. The decrease in PDI values indicates a narrowing of the molecular weight distribution and the formation of shorter chains caused by chain scission (Figure 8B). In addition, lower ***M_w_*** of the recycled polymer affects the mechanical performance of the material, which is characterized below.

### 3.5. Mechanical Properties

#### 3.5.1. Nanoindentation

Nanoindentation is a method used for the measurement of the local mechanical properties, such as the elastic modulus (***E***) and hardness (***H***) [37]. The range of microhardness values that each polymer exhibits is mainly determined by the nature of the molecular chains [38], which includes the structure, length, and polydispersity. As the recycling affected the structure, i.e., the length of the polymer chains, we used this technique to evaluate the effect of recycling on the micromechanical properties.

**Figure 9:**
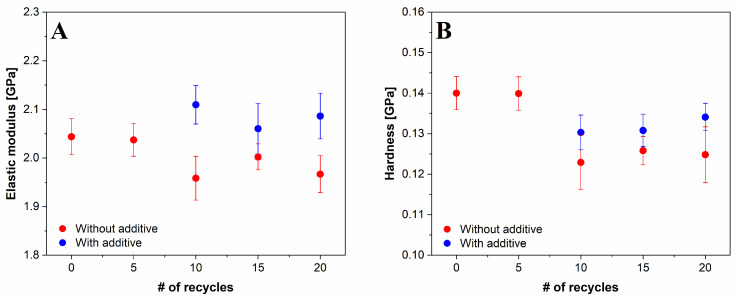
Average values of (**A**) elastic modulus and (**B**) hardness of virgin PP, recycled PP without and with additive. The average analysis depth was from 800 to 1800 nm.

The values of the elastic modulus (***E***) for the recycled PP samples, with and without additive, are presented in Figure 9A, while the values for surface hardness (***H***) are shown in Figure 9B. The average values of the elastic modulus and hardness are presented at a penetration depth of 800–1800 nm. The results show that after five reprocessing cycles, the ***E*** and ***H*** values remained the same; however, with further recycling, the ***E*** and ***H*** values decreased, indicating that the surface of the extensively recycled PP became more rigid. The results were found to be in close agreement with the work carried out by Zdiri et al. [39] and Bourmaud et al. [40], showing a slight reduction of elastic modulus after recycling, which could be due to physical ageing and thermo-oxidative degradation, i.e., decreasing of molecular weight [31]. On the other hand, the addition of an additive restores the elastic properties and hardness to higher values as detected for the virgin PP.

#### 3.5.2. Time-Dependent Behavior

**Figure 10:**
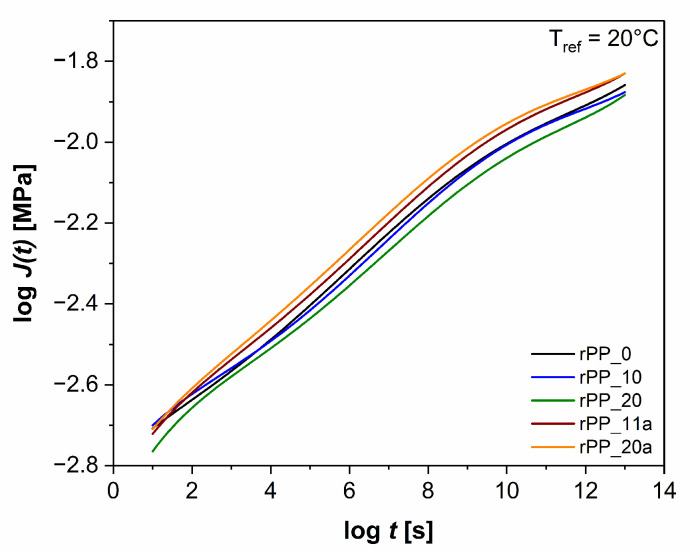
Creep master curve at the reference temperature (***T_ref_*** = 20 °C) for virgin PP, recycled PP without and with the additive.

The polymers suffer structural changes when used for a long time. However, exposure to natural operating conditions can last for a very long time before some observable changes occur. Hence, the accelerated procedures should be used for the prediction of long-term behavior of such materials. One of the possibilities to determine time-dependent mechanical properties of polymers is the use of a predictive method based on an increase in the test temperature, the so-called time-temperature superposition (***tTS***) principle [41]. The master curve of the long-term creep response can be composed of the short-term isothermal creep tests at different temperatures ***J***(***t***, ***T***) with an individual shifting of segments. In Figure 10, the average master curve of creep behavior (the dependence of the creep modulus, ***J***, on time, ***t***) of virgin and recycled PP without and with additive are presented. The results obtained from the creep modulus do not show any changes up to 10th recycle (rPP_10), but with further reprocessing cycles (rPP_20) the values of the creep modulus decreased compared to virgin PP. Furthermore, the addition of an additive increased the creep modulus to higher values, but the shape of the creep compliance curve did not change significantly. Creep tests showed that the addition of the additive improved the creep resistance of recycled PP in both time and temperature, with this benefit becoming more significant with increasing temperatures and longer times, respectively.

#### 3.5.3. Dynamic Mechanical Thermal Analysis (DMTA)

**Figure 11:**
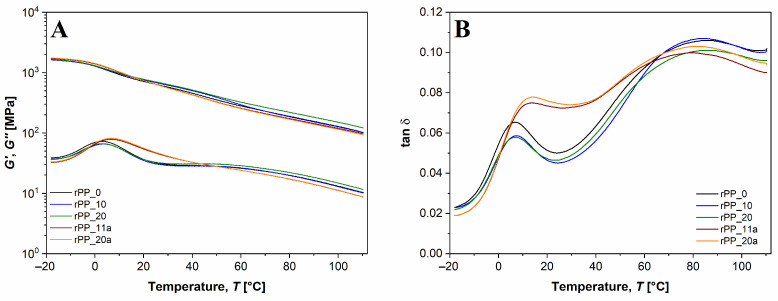
DMA curves: (**A**) storage modulus (***G’***) and loss modulus (***G’’***); (**B**) tan δ of virgin PP and recycled PP without and with the additive.

Dynamic mechanical thermal analysis (DMTA) is a widely used technique enabling the characterization of the mechanical behavior of materials as a function of temperature and/or frequency of oscillation. Figure 11A shows the temperature dependence of the storage modulus (***G’***) and loss modulus (***G’’***) of virgin and recycled PP without and with the additive. It can be seen that for all the samples, G’ was approximately one decade higher than ***G’’*** in the whole temperature range examined. The increasing temperature caused the softening of the PP matrix and increased molecular mobility [42]. At low temperatures, the PP recycled 20 times (rPP_20) showed the same values of ***G’*** and ***G’’*** as the virgin PP, but at temperatures higher than 60 °C, the values of the moduli for rPP_20 slightly prevailed over the values of virgin PP. The increase in ***G’’*** with extensive recycling of PP (rPP_20) leads to lower stiffness of the polymer. On the other hand, the recycled PP samples with the additive exhibited the lowest ***G’*** and ***G’’***, respectively, meaning that these samples became more flexible compared to virgin and recycled PP without the additive.

Tan δ is defined as the ratio between the loss and storage modulus (Tan δ = ***G’’***/***G’***) and is related to the damping properties of the polymer. The Tan δ curve of PP exhibits three relaxations peaks at about −80 °C (γ), 10 °C (*β*), and 100 °C (*α*). With our instruments the measurements could only be performed from the temperatures of −18 to 110 °C; therefore, the γ relaxation peak, which originates from the motion of the short segments in the amorphous phase, was too low to determine. In Figure 11B, the first peak within a temperature range from 0 °C to 20 °C corresponds to the glass transition temperature (***T_g_***) or *β* relaxation of PP [43]. ***T_g_*** of virgin PP was thus detected at 6.3 °C and this value remained constant up to the 20th reprocessing cycle. Furthermore, with the addition of the additive, ***T_g_*** increased to 13.5 °C. This indicates that the PP polymer chains in the amorphous region became more rigid. The ***T_g_*** values were obtained also from loss modulus curves, where the obtained values for rPP without additive were close to ~3 °C and ~7 °C for rPP_a samples, respectively. The second peak in the elastic-rubbery region, characteristic for *α* relaxation (long-range coordinated molecular motions), was located between 60 and 100 °C [44,45]. The *α* relaxation process for rPP_a samples occurred at lower temperatures (~81–82 °C) compared to rPP samples without the additive (85–88 °C). The ***T_g_*** values for the *β* and *α* relaxation process, determined from tan δ and loss modulus curves, respectively, are summarized in Table 4.

**Figure 12:**
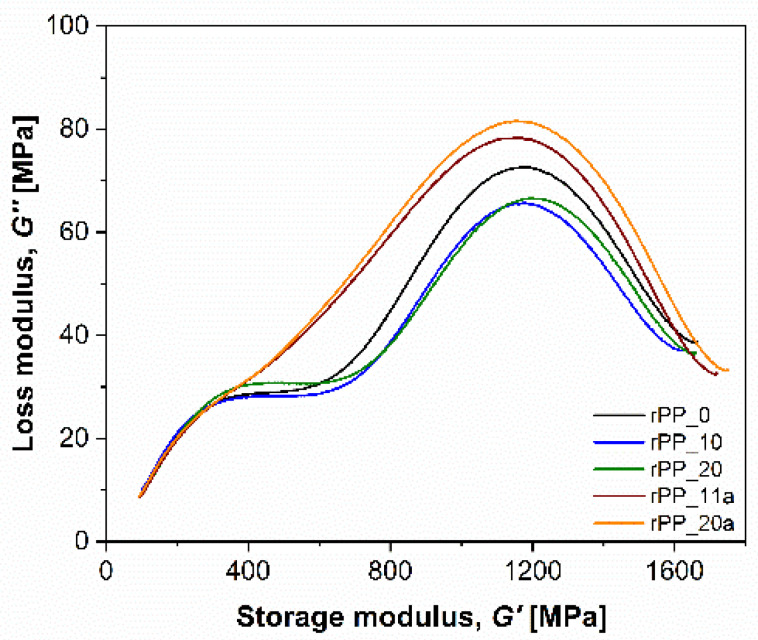
Cole–Cole plot of virgin PP and recycled rPP and rPP_a samples.

For studying the viscoelastic behavior, the Cole–Cole plots can provide additional information about the relaxation processes occurring by reprocessing polymers. In our study, the Cole–Cole plot showed the relationship between the loss modulus (***G’’***) and storage modulus (***G’***), and helped to understand the relaxation processes of a semi-crystalline PP polymer (Figure 12). For the virgin and recycled PP without additive, two peaks, observed at lower and higher temperatures, can be attributed to the ***β*** and *α* relaxation processes [46]. The first peak is related to the ***β*** relaxation process and could be ascribed to the segmental relaxation mechanisms in the amorphous region. This peak can also be related to glass transition (***T_g_***). The second peak, attributed to the *α*_c_ relaxation process, can be associated with relaxations appearing from the crystalline phases of the PP polymer matrix [45,47]. The addition of the additive to rPP (rPP_a), on the other hand, mimics the first peak and changed the shape of the Cole–Cole curves. The more pronounced second peak of the ***α*** relaxation process could be considered as an increasing exchange of stereo defects between the amorphous and crystal phases.

## 4. Conclusions

The extensive recycling of polymers leads to changes in the rheological, morphological, mechanical, thermal, and other properties of the processed polymer. In our study, it was shown that after 20 recycling steps of mechanical processing of polypropylene, thermo-oxidative degradation occurred. This was observed with spectroscopic measurements, which showed the appearance of an absorption band at 1735 cm^−1^. The addition of a stabilization additive led to the formation of new bands at 1575 and 1541 cm^−1^, suggesting interactions between the additive and the PP polymer.

The viscoelastic analysis showed that an increase in the frequency at which the polymer structure changes (***G’*** = ***G’’***) from liquid- to solid-like was observed for the extensive recycling of the additive-free samples, while the frequency of the crossover point, *ω*_*co*_, for the samples with the additive were lower compared to the virgin PP and only slightly changed with the extensive recycling. The increasing *ω*_*co*_ suggests that the molecular weight of the recycled polymer decreased. Lower molecular weight is associated with shorter molecules that disentangle faster and therefore, exhibit a higher crossover frequency, while longer molecules need more time to disentangle and exhibit a liquid-like behavior at lower frequencies. When the additive was added to the recycled PP polymer, the values of *ω*_*co*_ decreased, indicating that the molecular weight was higher compared to the virgin PP. The increasing slopes of the ***G’*** and ***G’’*** curves at low frequencies for the samples with the addition of the additive can be associated with a narrowing of the average molecular weight distribution. Furthermore, the results showed that when the additive was added to the recycled PP, the rheological properties were improved already after the first recycle (rPP_11a) and remained unchanged for the further ten recycling cycles.

The results of the thermal analysis revealed that the main mechanism of degradation during recycling of PP is the chain scission, as the thermograms did not show changes in the microstructure. With increasing recycling of the additive-free samples, the increase in crystallinity for the samples from the 10th to the 20th recycle was much higher (~5%) compared to the samples with the additive (~0.2%). We can conclude that shorter chains acted as nucleating agents causing easier formation of crystalline regions. These results were also confirmed by the POM technique.

The narrower distribution of polymer molecules after the addition of the additive can also be associated with the improved mechanical properties of the surface, determined by nanoindentation measurements. The results showed that the additive-free rPP samples exhibited a slightly lower elastic modulus and hardness than rPP_a samples, suggesting a hardening of the surface during extensive recycling without the additive.

The DMA analysis was used to determine the ***T_g_*** of the PP and rPP samples. The results showed two tan δ peaks, the first which was for the virgin and recycled rPP without additive close to ~7 °C and was attributed to *α* relaxation (long-range coordinated molecular motions). For recycled rPP with additive, this peak was observed at higher values, i.e., ~13.5 °C. However, the *β* relaxation process for rPP_a samples occurred at lower temperatures (~81–82 °C) compared to the samples without the additive (85–88 °C).

The results showed that the use of an additive improves the time-dependent mechanical properties of solid polypropylene since it was shown that the additive acts as a hardener and additionally crosslinks the recycled polymer chains. On the other hand, the decrease in creep compliance modulus indicates a shorter lifespan with an increasing number of recycles due to chain scission of polymer chains. In summary, the main degradation processes of polypropylene that occur after extensive recycling are chain scission of polymer chains and oxidation, which, however, can be improved by the addition of a stabilization additive.

## Figures and Tables

**Table 1:** Sample classification and corresponding abbreviations.

Sample	Abbreviation
Virgin PP	rPP_0
5× reprocessed PP	rPP_5
10× reprocessed PP	rPP_10
15× reprocessed PP	rPP_15
20× reprocessed PP	rPP_20
11× reprocessed PP with additive	rPP_11a
15× reprocessed PP with additive	rPP_15a
20× reprocessed PP with additive	rPP_20a

**Table 2:** Process parameters for injection-molding of test bars for DMA.

Sample	Abbreviation
Melt temperature (Tm)	190 °C
Waiting time	3 min
Mold temperature	50 °C
Holding pressure	500 bar
Injection time	10 s
Post-pressure	100 bar
Post-processing time	10 s
Melt temperature (Tm)	190 °C

**Table 3:** Thermal properties, obtained from the DSC of virgin PP, recycled PP with and without additive.

	1st Heating		Cooling		2nd Heating		
# of Recycles	*H_m_* (J/g)	*T_m_* (°C)	*H_c_* (J/g)	*T_c_* (°C)	*H_m_* (J/g)	*T_m_* (°C)	*X_c_* (%)
rPP_0	99.0	166.0	100.9	112.9	105.7	162.0	51.1
rPP_5	99.5	166.1	101.8	120.2	106.4	163.3	51.4
rPP_10	99.7	165.7	103.1	120.4	108.2	163.5	52.3
rPP_15	98.8	166.1	103.6	121.2	108.5	163.0	52.4
rPP_20	94.0	165.5	102.3	121.5	106.7	162.5	51.3
rPP_11a	91.0	165.1	95.5	120.1	99.6	162.9	48.1
rPP_15a	91.2	165.2	96.8	120.6	101.1	163.3	48.8
rPP_20a	91.3	165.3	97.1	120.9	101.7	163.3	49.1

**Table 4:** Dynamic mechanical analysis (DMTA) results of virgin PP and recycled PP without and with the additive.

Sample	*T_g_* (°C) ^1^	*T_g_* (°C) ^2^	α Relaxation Peak (°C)
rPP_0	6.3	2.5	86.7
rPP_10	7.5	3.2	84.5
rPP_20	6.7	3.0	86.7
rPP_11a	13.5	6.2	81.0
rPP_20a	13.5	6.7	82.0

^1^*T_g_* obtained from *tan δ*; ^2^ *T_g_* obtained from storage modulus *(G’).*

## Data Availability

Not applicable.
